# The In-Depth Studies of Pulsed UV Laser-Modified TiO_2_ Nanotubes: The Influence of Geometry, Crystallinity, and Processing Parameters

**DOI:** 10.3390/nano10030430

**Published:** 2020-02-28

**Authors:** Łukasz Haryński, Katarzyna Grochowska, Piotr Kupracz, Jakub Karczewski, Emerson Coy, Katarzyna Siuzdak

**Affiliations:** 1 Centre for Plasma and Laser Engineering, The Szewalski Institute of Fluid-Flow Machinery Polish Academy of Sciences, Fiszera 14 St., 80-231 Gdańsk, Poland; lharynski@imp.gda.pl (Ł.H.); kgrochowska@imp.gda.pl (K.G.); pkupracz@imp.gda.pl (P.K.); 2 Faculty of Applied Physics and Mathematics, Gdańsk University of Technology, Narutowicza 11/12 St., 80-233 Gdańsk, Poland; jakub.karczewski@pg.edu.pl; 3 NanoBioMedical Centre, Adam Mickiewicz University, Wszechnicy Piastowskiej 3 St., 61-614 Poznań, Poland; coyeme@amu.edu.pl

**Keywords:** TiO_2_ nanotubes, laser treatment, phase transformation, surface modification

## Abstract

The laser processing of the titania nanotubes has been investigated in terms of morphology, structure, and optical properties of the obtained material. The length of the nanotubes and crystallinity, as well as the atmosphere of the laser treatment, were taken into account. The degree of changes of the initial geometry of nanotubes were checked by means of scanning electron microscopy, which visualizes both the surface and the cross-section. The phase conversion from the amorphous to anatase has been achieved for laser-treated amorphous material, whereas modification of calcined one led to distortion within the crystal structure. This result is confirmed both by Raman and grazing incident XRD measurements. The latter studies provided an in-depth analysis of the crystalline arrangement and allowed also for determining the propagation of laser modification. The narrowing of the optical bandgap for laser-treated samples has been observed. Laser treatment of TiO_2_ nanotubes can lead to the preparation of the material of desired structural and optical parameters. The usage of the motorized table during processing enables induction of changes in the precisely selected area of the sample within a very short time.

## 1. Introduction

Among many n-type semiconductors, titanium dioxide nanotubes (TiO_2_NTs) have gained a lot of attention during the last decades due to their chemical stability, photocorrosion resistance, or biocompatibility [1,2]. Moreover, it should be underlined that their semiconducting properties strongly depend on morphology as highly ordered tubular architecture ensures a large internal active area, facilitates charge transport along the tube and enhances light absorption [1]. Nanotubes (NTs) can be formed by applying three different routes: (i) template-based approach [2], (ii) hydrothermal synthesis [3], and (iii) anodization process [4]. However, due to the simplicity and low-cost of the experimental setup, anodization seems to be the most appropriate method to obtain the NTs [5]. Such an approach allows for the precise control over the geometry of the NTs by manipulation of the processing parameters, such as solution content, time, temperature, or stirring speed. The very first formation of porous anodic oxide film on titanium substrate was achieved in fluorine ions-containing electrolyte and reported by Zwilling et al. [6]. The nanotube formation during the anodization can be explained according to the mechanism described by Galstyan et al. [7]. In general, during the anodization the tubular geometry is being achieved as the result of balance between two processes: electrochemical oxidation of titanium and chemical dissolution of the formed oxide layer by F^-^ ions. Initially, the TiO_2_ layer grows at the interface between Ti and an electrolyte according to the Equation (1):

Ti^4+^ + 2H_2_O → TiO_2_ + 4H^+^. (1)

Afterwards, the as formed oxide layer is being chemically dissolved, forming at first cracks and pits in the oxide layer. The chemical reaction responsible for dissolution is described by the Equation (2):

TiO_2_ + 6 F^−^ + 4H^+^ → [TiF_6_]^2−^ + 2H_2_O. (2)

The migration of F^-^ ions towards positively charged electrode (at this stage, it is Ti covered by a dense TiO_2_) is enhanced by the external voltage. Initially formed cracks are rapidly dissolved regarding surroundings due to the continuous diffusion of the F^-^ ions. Hereby, the cracks are transformed into porous nanoarchitecture. However, as the anodization proceeds, the areas between the pores are gradually etched by F^-^ ions and ordered nanotubes arise. Nevertheless, as-obtained nanotubes exhibit amorphous phase and calcination is usually performed to obtain crystalline phase [8]. According to the literature [9], in the calcined TiO_2_NTs two crystalline phases, anatase and rutile, can be found. The particular anatase-to-rutile ratio depends on the applied temperature and could vary from 60 to 15% for samples annealed at 400 and 550 °C, respectively.

Taking into account heating up and cooling down periods the whole process of the traditional furnace treatment can take over 10 h. Therefore, many different strategies are being developed to shortened time or decrease temperature needed for inducing the crystallization of TiO_2_NTs. For example, Liao et al. [10] proved that the phase conversion can be achieved without heating up to high temperatures as TiO_2_ in the form of nanotubes or powders can be crystallized by immersing the samples in water at 92 °C for 35 h. Moreover, Wang et al. [11] reported the phase conversion of the as-anodized TiO_2_NTs into anatase by soaking them in the water at room temperature. Nevertheless, their morphology has changed from ordered tubes into mesoporous nanowires. Interestingly, after transformation, the surface area has increased about 5.5 times in comparison to the initial surface of NTs. Another example of water-induced phase conversion was presented by Lamberti et al. [12] as NTs arrays exposed to the water vapor underwent complete phase conversion in ca. 30 min. Recently, even faster approaches have been studied, namely laser annealing and plasma treatment. In the case of the latter, Benčina et al. [13] crystallized TiO_2_NTs in few seconds by means of highly reactive oxygen plasma, without affecting the morphology. As it comes to laser annealing, it has been so far used for phase conversion of different TiO_2_ morphologies, mostly thin coatings. Joya et al. [14] were able to obtain Ag-TiO_2_ nanocomposite accompanied by anatase crystallization as a result of laser irradiation of films formed by titania precursors. Moreover, it was reported that the addition of Ag improved the phase conversion during the processing. Overschelde et al. [15] transformed initially amorphous magnetron sputtered TiO_2_ nanofilm into crystalline one by applying UV excimer laser irradiation. Nevertheless, the process led to the complete change of the morphology as the formation of spherical nanoparticles occurred. The influence of iron and aluminium onto the UV laser-induced anatase to rutile transformation of titania nanoparticles was reported by Vásquez et al. [16]. It has been found that Fe-doping promotes the phase conversion while Al-one inhibits it. It can be easily seen that laser treatment has been successfully applied for the phase transformation of various titania material. However, in the literature, there are very few reports on laser annealing of TiO_2_NTs. In 2008, Likodimos et al. [17], for the very first time reported local structural transformation of amorphous TiO_2_NTs to anatase phase by means of laser from Raman spectrometer. The vibrational modes of crystalline anatase after exposing of as-anodized samples to the highest laser power followed by subsequently scan at low laser power were observed. On the contrary to thermal annealing, graphitic band at 1592 cm^−1^ with a broad shoulder at ca. 1400 cm^−1^ was observed on laser annealed Raman spectra. This phenomenon was explained by organic ions trapping in the anodic matrix which in case of thermal annealing is converted to volatile compounds, e.g., carbon dioxide. Hsu et al. [18] investigated structural and morphological transformations of amorphous TiO_2_NTs after excimer pulsed-laser irradiation. Samples were mounted at the fixed angle to the laser beam and crystallinity up to 90% of laser annealed samples compared to furnace annealed ones at 400°C for 1 h was reported. However, the depth of the phase conversion was not established. Enachi et al. [19] examined the impact of 532 nm continuous laser on the morphology and crystallinity of as-anodized TiO_2_NTs. Following this approach they observed anatase or anatase/rutile mixed phases within a laser spot area. Nevertheless, as the laser spot was not homogenous and its shape was not strictly defined, the proposed route cannot be used for further purposes. Moreover, since Raman spectra recorded from laser annealed samples were not compared with calcined ones the results did not provide full information about the process. Xu et al. [20] modified calcined titania NTs by means of pulsed 248 nm laser, and the irradiation under optimum conditions resulted in enhanced photoelectrochemical response. However, that approach is limited to the small samples due to the requirement of their immersion in deionized water and their rotation with a motorized actuator to homogenize area of modification.

Taking into account the results obtained so far, the laser treatment can be considered as the most promising alternative for furnace calcination. As it comes to traditional thermal annealing, below 200 °C, the amorphous phase is maintained for TiO_2_ nanotubes and with increased temperature and time of processing the crystallinity (anatase phase content) is increasing [21]. From 450 °C, the rutile phase is being more pronounced. Overall, temperature of 450 °C seems to be the optimal one taking into account the crystallinity and small amount (ca. 5%) of rutile. The main advantage of the calcination is then the fact that the whole sample undergoes processing at the same time. However, it should be kept in mind that at least 1–2 h are needed to crystallize the nanotubes and prolonged treatment can lead to formation of cracks in the structure. It should be also remembered that, taking into account time needed for furnace heating and cooling, the whole traditional thermal treatment lasts over a dozen h. Moreover, most of the heat is transported to the nanotubes both from substrate foil and the boat or plate used as a platform during thermal treatment. Taking into account above and the fact that thermal conductivity of TiO_2_ is lower than the one of Ti, rutile is more likely to be formed [22], at the TiO_2_/Ti interface. Additionally, due to different thermal response of TiO_2_ nanotubes and Ti foil, material interface sliding, structure degrading, efficiency downgrading and mechanical failure can occur [23], meaning that the main advantage could be also considered as the biggest disadvantage. On the other hand, the main advantage of the laser treatment is its rate – the crystallization of the nanotubes takes from only few seconds up to few min. Moreover, the temperature remains more or less uniform within the whole processed area (nevertheless, for more uniformity additional optical element, namely laser beam homogenizer can be used). Another advantage of the laser processing is its tunability. By changing working laser parameters one can obtain the desired ratio of anatase and rutile [14]. Moreover, as the laser interact with nanotubes from the top, the fabrication of crystalline features onto low-thermostable substrates can be achieved (in case when TiO_2_ are not attached to the Ti foil) [24]. By applying different masks or when the material is attached to the motorized table or just simply to production tape, it is possible to crystallize samples of desired shape and size. The main disadvantage though is related to the fact that during processing the thermal conductivity of the titania material is being changed that can lead to melting or ablation. Finally, lasers are popularly applied in the commercial production; thus, the process up-scaling does not pose a lot of difficulty.

In this work, we continue our studies over laser treatment of titania nanotubes. In our previous report [25], the amorphous sample was kept in an ambient atmosphere while the idea of the research was to find application for shadow-mask assisted laser modification. The impact of the nanotubes length or atmosphere conditions onto the morphology, structural and optical properties was not considered. Regarding another work that we referred to here [26], we focused only on the laser treatment of the already calcined titania and investigated the effects onto the photoelectrochemical activity. On the contrary, herein we focus onto the different titania substrates used for further laser modification: amorphous and calcined ones while the laser treatment has been carried out both in air and under vacuum conditions. The undertaken efforts were focused to track the impact of proposed approach onto morphology and optical properties: light absorption and generated photoluminescence. What is of high importance and till now has not been shown, is the presentation of crystalline phase investigation with the use of grazing incident XRD (Gi-XRD) indicating the crystallinity degree along the tubular layer. Therefore, in here, we report on the effect of laser treatment onto the properties of amorphous and crystalline NTs irradiated both in vacuum and in the air. Scanning electron microscopy, photoluminescence, XRD diffractometry, UV-Vis and Raman spectroscopies were utilized for the in-depth investigation of the prepared material. Obtained results and the usage of the motorized table during processing strongly suggest that laser-treated TiO_2_NTs can be used in the applications enabled by their optical properties, and the process can be easily scaled up to the commercial level.

## 2. Materials and Methods

Prior to the anodization, Ti foil (Strem, 99.7%) was ultrasonically cleaned in acetone, ethanol, and water for 10 min in each solvent, followed by drying in the air. The anodization process was realized in a two-electrode setup, where the titanium plate served as an anode and the platinum mesh as a cathode with a fixed distance of 2 cm between them. Highly ordered TiO_2_NTs were prepared via one-step anodization at 40 V for 15, 30, 45, and 60 min in an electrolyte containing ethylene glycol (EG), 0.22 M NH_4_F, and 15 vol. % of deionized water. The temperature during the reaction was set to 23 °C and maintained by the thermostat working in the flow-mode. After the anodization, the samples were rinsed with ethanol and then dried in the air. According to the anodization time the samples were labelled as TiO_2_-X, where X stands for the time of anodization. Finally, selected samples were thermally annealed at 450 °C for 2 h, with a heating rate of 2 °C/min. Laser annealing of prepared material was performed by means of 355 nm Nd:YAG pulsed laser (Quantel) equipped with the beam homogenizer at 2 Hz repetition rate in order to minimize the accumulation of thermal energy in the sample. The energy fluence was set to 25 mJ/cm^2^ and during laser irradiation samples were mounted on the motorized table with a constant speed of 3.5 mm/min. The process was performed in the air and in the vacuum chamber under 5 × 10^−5^ mbar pressure. All samples prepared for particular processing parameters, namely anodization time, thermal treatment, and atmosphere, during laser treatment are listed in Table 1.

The surface morphology and geometry of nanotubes were examined using the Schottky field emission scanning electron microscopy (FE-SEM, FEI Quanta FEG 250) with an Everhart-Thornley (ET) secondary electron detector. The voltage was kept at 10 kV.

Grazing incident X-ray diffractometry (Gi-XRD) experiments were performed by a PANalytical X’pert^3^ MRD diffractometer, working with Cu- Kalpha radiation, operating at 45 mA and 40 kV and equipped with a point scintillator detector. Experiments were carried out with angle increments and time per step of 0.02° and 15 s, respectively. Incident angle (ω) was calculated according to the estimated X-Ray attenuation length [27] and SEM micrographs of the samples. Scan size was selected to detect the main diffraction peaks of known TiO_2_ crystalline phases (anatase, brookite, and rutile), from 10 to 40°. It is important to remark, that samples were not perfectly flat foils and that laser-treated areas were the central sections of a larger substrate. Therefore, the absolute surface was difficult to determine, especially when considering the possibility of amorphous surfaces. Nevertheless, by slightly tilting the goniometer towards the incident beam (fixed at 0.05° for this purpose) and then scanning the Z-axis, with the detector positioned at the substrate peaks, the untreated end of the sample could be detected. Finally, experiments were performed after removing the tilting correction and keeping substrate peaks at a similar intensity. Notice that all samples showed a slight concave bend of the Ti foils; thus, the determination of an approximated nanotube’s surface was achieved in this way.

The UV-Vis reflectance spectra of TiO_2_NTs were measured with a dual-beam UV-Vis spectrophotometer (Lambda 35, Perkin-Elmer) equipped with a diffuse reflectance accessory. The spectra were recorded in the range of 300–1100 nm, with a scanning speed of 120 nm/min.

The Raman spectra were recorded by a confocal micro-Raman spectrometer (InVia Renishaw) with sample excitation by means of an argon ion laser emitting at 514 nm and operating at 10% of its total power (50 mW).

The photoluminescence (PL) spectra were recorded using a laboratory setup consisting of a 0.3 m Czerny-Turner spectrograph (SR303i, Andor) equipped with an intensified charge-coupled device (ICCD) camera (DH740, Andor) and UV LED (365 nm center wavelength, 9 nm FWHM (full width at half maximum), 350 mW output power). The excitation radiation was focused by quartz lens and reached the sample surface at an angle of 45°. In order to block the UV LED radiation above 380 nm, the band-pass filter (UG11, Schott) was applied. The signal was collected using a microscope objective perpendicular to the sample surface and focused on the entrance of the optical fiber. Band-pass filters (GG44, Schott) were used for blocking the excitation radiation in the detecting path.

## 3. Results and Discussion

**Figure 1:**
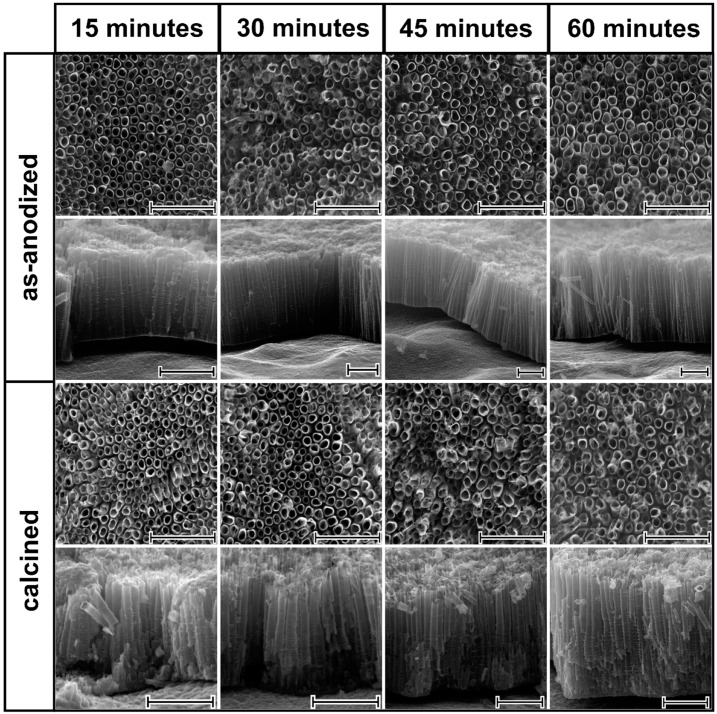
Top and cross-section SEM images of as-anodized and calcined titanium dioxide nanotubes (TiO_2_NTs) obtained for different anodization times, namely 15, 30, 45, and 60 min. Scale bars are set to 1 µm.

**Figure 2:**
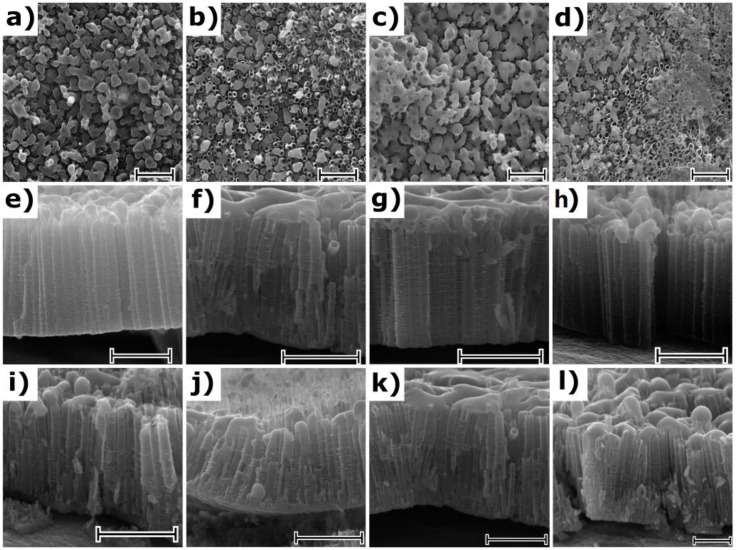
Top and cross-section SEM images of laser-modified samples under different atmospheres: (**a**,**e**) as-anodized TiO_2_-45 in air, (**b**,**f**) calcined TiO_2_-45 in air, (**c**,**g**) as-anodized TiO_2_-45 in vacuum, (**d**,**h**) calcined TiO_2_-45 in vacuum, (**i**) calcined TiO_2_-15 in air, (**j**) calcined TiO_2_-30 in air, (**k**) calcined TiO_2_-45 in air, and (**l**) calcined TiO_2_-60 in air. Scale bars are set to 1 µm.

SEM images of as-anodized aligned TiO_2_NTs are shown in Figure 1. As can be easily seen, the tubular structure was reached for anodization time that lasts for 15 min. This indicates that the steady state, where electrochemical oxidation and chemical dissolution rates are equal, was reached during the formation of TiO_2_NTs. According to the literature [4], prolonged anodization time may result in bottom up narrowing of the NTs wall, since the electrochemical oxidation takes place at the Ti/TiO_2_ interface while more intensified etching occurs at the outer NTs layer. Considering cross-sections for the TiO_2_NTs obtained from 60 min anodization, some insignificant difference in wall thickness of NTs at the base and at the surface region can be observed. It is worth emphasizing that wall etching, reported in the literature for the prolonged anodization times [8], was not observed for any sample. It can be noted that, the internal diameter and wall thickness of all obtained titania samples do not change significantly regardless of the anodization time and they are estimated to be 90 ± 10 nm and 10 ± 3.5 nm, respectively. The length of the pristine TiO_2_NTs reaches 1.5, 2.4, 2.5, and 3.2 µm for anodization times of 15, 30, 45, and 60 min, respectively. The impact of the laser radiation on the as-prepared and calcined titania nanotubes is presented in Figure 2. Despite the chosen energy fluence was very low, the re-solidified layer that is covering the top of the surface of the nanotubes was present irrespective of the atmosphere of the laser processing. Comparing the SEM images for the as-anodized and calcined samples treated by laser, it can be concluded that the mentioned re-solidified layer is less pronounced for the latter ones, which is probably related to the better thermal conductivity of the calcined samples. Moreover, during the laser annealing in air, one should take into account the heat transfer between the NTs and the surrounding gas molecules, which could lower the surface temperature, thus causing changes in the geometry. Nevertheless, it should be also noted that the tubular structure underneath was intact for all modified TiO_2_NTs and is still fixed at the Ti substrate. The melted layer thickness of the nanotubes dependence on their length and the type of atmosphere was not observed. The thickness of the re-solidified layer is ca. 300 nm for all samples, and the same observation was reported by Xu et al. [20].

**Figure 3:**
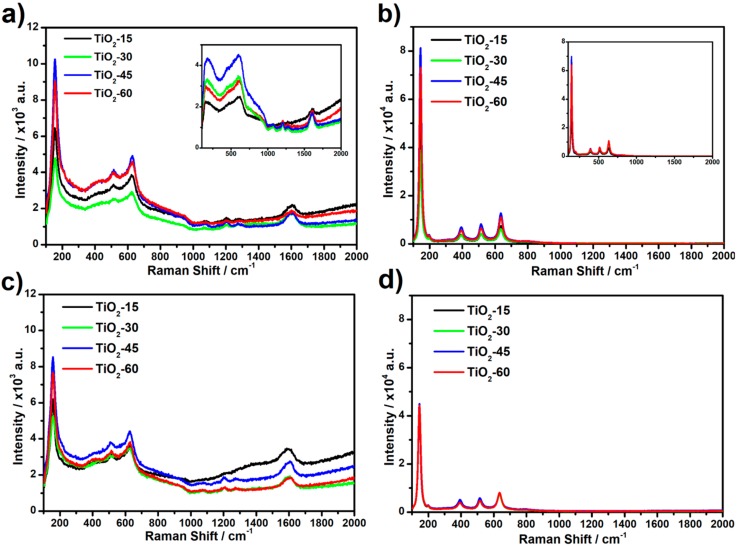
Raman spectra recorded for laser modified TiO_2_NTs: (**a**) as-anodized in air, (**b**) calcined in air, (**c**) as-anodized in vacuum, and (**d**) calcined in vacuum. Insets presented in Figure 3a,b represent unmodified titania samples: amorphous and calcined, respectively.

Raman spectra recorded for laser annealed titania samples, as well as for reference ones, are shown in Figure 3. Signals recorded for as-anodized TiO_2_NTs (see inset, Figure 3a) at ca. 200, 450, and 600 cm^−1^ correspond to the amorphous Ti-O bending and stretching vibrations [28], while the ones observed at ca. 1040, 1070, 1200, and 1270 cm^−1^ can be assigned to the liquid glycol remaining in titania pores [29]. Therefore, it can be concluded that cleaning of the NTs in ethyl alcohol after anodization is not effective and some residual electrolyte molecules could be present on the surface of the samples. Moreover, the signal observed at ca. 1600 cm^−1^ corresponds to the graphitic G band in disordered carbon materials which is likely related to the presence of amorphous carbon [30]. Signals recorded for calcined titania samples (see inset, Figure 3b) are located at 144, 197, 394, 515, and 635 cm^−1^ and are related to the E_g(1)_, E_g(2)_, B_1g_, A_1g_, and E_g(3)_ active anatase modes, respectively [31]. Spectra obtained for laser modified as-anodized samples contain only three active anatase modes: E_g(1)_ at 156, A_1g_ at 510 and E_g(3)_ at 632 cm^−1^ and it can be suggested that the NTs are not completely converted from amorphous to anatase phase. Such a mode shift of E_g(1)_ from 144 cm^−1^ to 156 cm^−1^ observed for laser-annealed as-anodized samples in relation to furnace calcined ones is likely related to the formation of oxygen vacancies during the process [32]. The bands corresponding to glycol, as well as amorphous carbon molecules, are also present. Additionally, no significant difference between the intensity of Raman signal recorded for laser-processed as-anodized TiO_2_NTs in air and under vacuum is observed. In the case of the laser modified calcined samples, the position of the bands has not changed comparing to the calcined non-treated ones whereas intensity decrease dramatically for samples modified in vacuum. Possible degradation of the crystal structure for calcined samples treated in vacuum is probably related to the heat accumulation at the top of the NTs caused by limited heat exchange with the surroundings in comparison to ones modified in air. Due to the preceding thermal treatment in furnace, no bands corresponding to glycol nor amorphous carbon are present. Moreover, no peaks originating from rutile modes are observed for all obtained samples.

**Figure 4:**
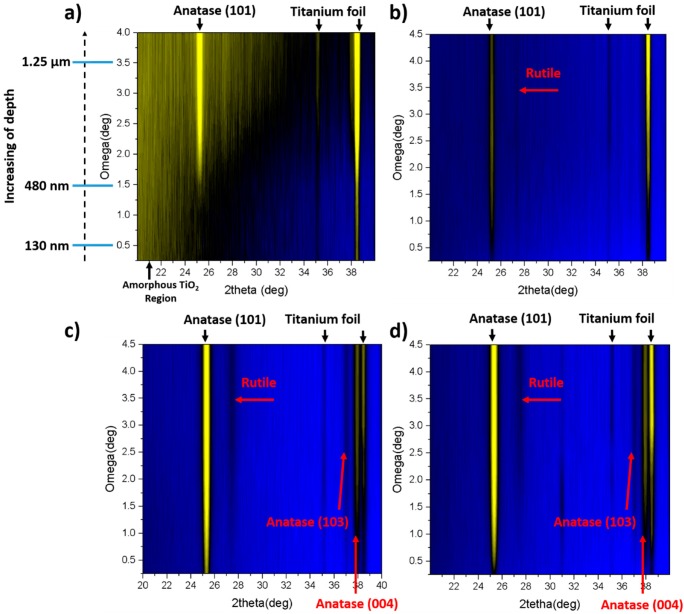
Grazing incident XRD (Gi-XRD) 2-D image scans of amorphous (**a**,**b**) and calcined (**c**,**d**) TiO_2_-45 nanotubes annealed by laser in air (**a**,**c**) and under vacuum (**b**,**d**).

For grazing incident XRD measurements, the TiO_2_NTs formed in the anodization process that lasts 45 min are chosen as they exhibit the most intensive Raman signals. The results for amorphous and calcined TiO_2_-45 nanotubes treated by laser irradiation both in air and under vacuum conditions are shown in Figure 4. For the as-anodized sample annealed in air it can be observed that at the top of the NTs amorphous phase can be still detected (Figure 4a). According to the appearance of anatase signal, the length of the amorphous region reaches ca. 480 nm. Nevertheless, with the increasing depth alongside the tube, the anatase (101) signal becomes more pronounced. That observation is in agreement with Raman data as the mixture of anatase and amorphous phases can be seen in Figure 3a. The presence of surface amorphous phase could result from the rapid melting accompanied by resolidification without achieving the crystalline phase. Furthermore, Hsu et al. [18] also reported on the presence of anatase (101) for the amorphous TiO_2_NTs treated by KrF excimer laser with 67 mJ/cm^2^ fluence. Nevertheless, it was stated that with the increasing value of laser energy, the rutile peaks appear in XRD patterns. That is not observed in here, as the chosen energy fluence is high enough only to overcome the nucleation barrier for the transformation of TiO_2_NTs into anatase [26]. However, when the same sample is processed in vacuum (Figure 4b), no amorphous region is observed and anatase (101) peak can be seen at a penetration depth of ca. 10 nm. It can be also concluded that the crystallinity of the material increases with the depth of penetration. Moreover, the weak peak corresponding to rutile also appears at Gi-XRD 2-D image scan that is not measured by means of Raman spectroscopy. The presence of rutile phase for the amorphous TiO_2_NTs processed in vacuum can be explained by the well-known fact that heat transport in vacuum is much worse than in air; therefore, the crystallization is more efficient in this environment for the same laser fluence.

**Figure 5:**
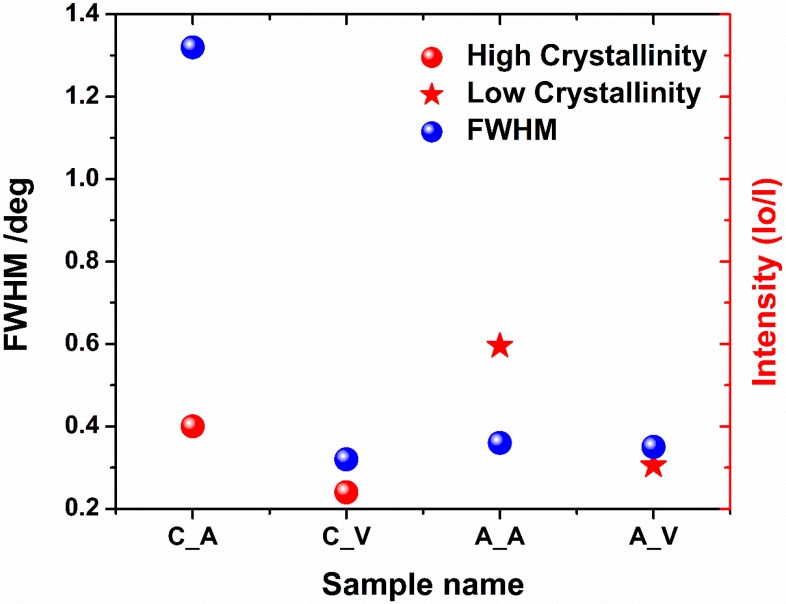
The ratio between intensities of anatase and substrate peaks for amorphous (A_A, A_V) and calcined (C_A, C_V) TiO_2_-45 nanotubes laser-treated in air (A_A, C_A) and under vacuum (A_V, C_V) conditions. For the reason of mathematical logic, for calcined samples Io states for intensity of anatase and I—for intensity of the substrate, while for amorphous ones—vice versa.

In the case of calcined TiO_2_ material, the anatase (101) peak is clearly seen for samples treated both in air and under vacuum (see Figure 4c,d), and it can be detected from the depth of ca. 5 nm. Like in the case of the amorphous TiO_2_NTs, the crystallinity degree of the samples enhances with the penetration depth. Additionally, a small amount of rutile can be observed and higher-ordered peaks for anatase, namely (103) and (004) are also visible. Comparing all Gi-XRD 2-D images, as well as the estimated ratio of intensities of anatase and substrate peaks (see Figure 5), it can be stated that the calcined samples processed by laser exhibit higher intensity and crystallinity regarding the amorphous ones. That means that the laser beam fluence was low enough not to degrade the crystal structure of the samples. Nevertheless, it can be clearly seen that the ratio is smaller for a calcined sample treated in vacuum in comparison to the one treated in the air, which suggests that the degradation process may be initiated, and this observation is in agreement with the Raman measurements (see Figure 3b,d).

**Figure 6:**
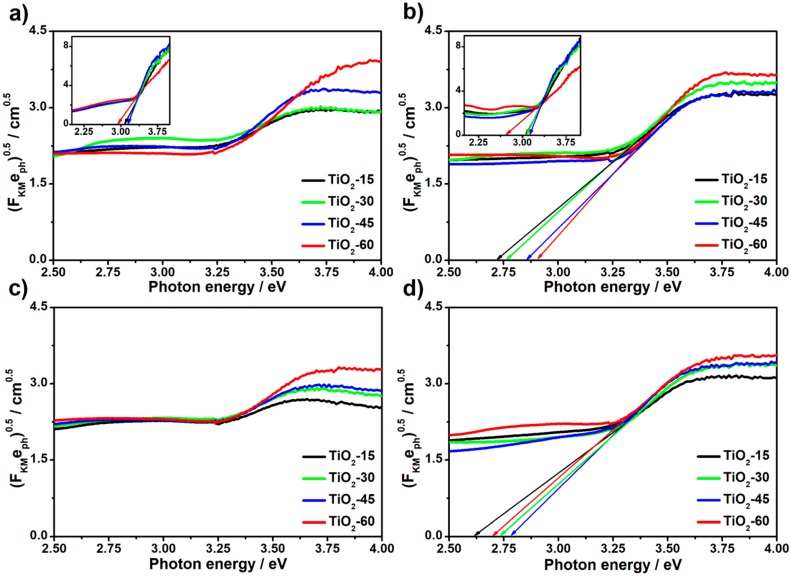
Tauc plots for as-anodized (**a**,**c**) and calcined (**b**,**d**) TiO_2_NTs laser-processed in the air (**a**,**b**) and under vacuum (**c**,**d**). Insets in Figure 6a,b represent unmodified as-anodized and calcined samples, respectively.

The influence of laser processing, as well as the atmosphere conditions on the optical properties of all obtained titania material, was investigated by means of UV-Vis spectroscopy. To determine the optical bandgaps, Tauc plots were derived from reflectance data and they are shown in Figure 6. The values of bandgaps for laser-modified titania samples, as well as for unmodified, reference ones, are presented in Table 2. Due to the low crystallinity degree of the laser-treated as-anodized TiO_2_NTs (see Figure 5), the values of bandgaps was not determined. It can be seen that the values for reference as-anodized samples are decreasing with the prolonged anodization process and thus with the increasing length of the nanotubes (compare TiO_2_-15 and TiO_2_-60). This can be attributed to the more pronounced aggregation of the vacancies, which can act as trap states alongside the tubes and finally lead to the lowering of the band-to-band transition energy [33]. The same trend is preserved for reference crystalline TiO_2_NTs and the bandgap values do not change significantly in comparison to the as-anodized material. This can be related to the fact that both anatase and amorphous titania exhibit similar electronic structure [34]. It is worth mentioning that estimated values for anatase samples are slightly lower than 3.2 eV which is typically reported for anatase phase in bulk [35]. Since the optical properties of the TiO_2_NTs depend on the geometric features of tubular structure [33], the difference most likely results from the specific morphology of the calcined sample.

In the case of laser annealed crystalline TiO_2_ nanotubes, the narrowing of the energy band gap can be easily observed. This phenomenon can be related to the introduction of additional states, so-called “shallow donors”, resulting from the degradation of the crystal structure [20]. However, on the contrary to the as-anodized samples, the values of bandgaps for calcined TiO_2_NTs modified by laser rise with the length of the nanotubes. Moreover, the narrowing of the bandgap is more distinct for the vacuum-treated samples possibly due to the further degradation of the anatase structure which is consistent both with Raman and XRD data.

On the other hand, one can observe that laser-modified TiO_2_NTs exhibit significantly lower absorbance in the UV region comparing to unmodified ones. This could be attributed to the presence of remelted surface which act as a mirror for light from this wave range. Furthermore, it can be observed that the absorption band edges are not as sharp for laser-treated samples as for the unmodified ones. It can be related to the oxygen deficiency and thus the presence of Ti^3+^ ions [36] for laser-treated material. It is also worth noticing that, for samples processed in the air, the edges are sharper than for ones obtained in vacuum suggesting the more degree of oxygen deficiency and, therefore, the higher number of Ti^3+^ ions in latter ones.

**Figure 7:**
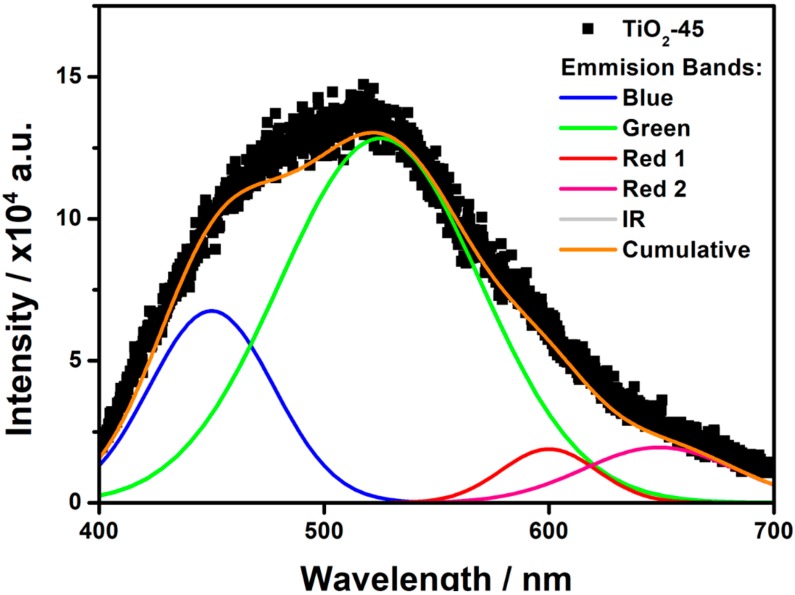
The emission spectrum of as-anodized TiO_2_ nanotubes obtained for the 45-min synthesis.

To investigate the separation of photoinduced charge carriers, as well as the surface oxygen vacancies and defects, the photoluminescence measurements were conducted. The PL spectrum of the as-anodized TiO_2_-45 sample is shown in Figure 7. The broadband shows the main maximum at around 520 nm with a strong shoulder on the low wavelength side and a long tail on the other. Taking into account resolved PL spectra reported by other authors [37,38,39,40,41] the distinctive emission bands should be founded at around λ_blue_ ≈ 400–480 nm, λ_green_ ≈ 510–530 nm, and λ_red_ ≈ 600–650 nm. The green PL peak is related to the recombination of mobile (conduction or shallow-state) electrons with trapped holes. On the other hand, Pallottti et al. [41] have shown that the green PL emission undergoes reduction under the presence of oxygen absorbed on a surface of the TiO_2_NTs. This effect is observed because chemisorption of superoxide species (O_2_^−^) leads to the subtraction of photoelectrons from the conduction band, as well as enlarging the depth of the surface depletion layer and shrinking the region in which photoelectrons can recombine with PL-active defects. Therefore, an increment of green PL emission of amorphous samples due to the laser treatment may be a combined effect of a growing number of trapped holes and a decrement of the specific surface during the laser treatment.

**Figure 8:**
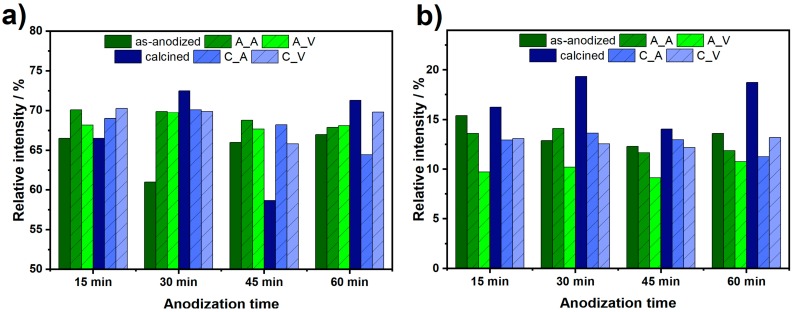
The relative area of the different emissions: (**a**) green, (**b**) red for laser annealed titania nanotubes, as well as references: as-anodized and calcined. Shortcuts: A_A, A_V, C_A, C_V mean as-anodized modified in air, as-anodized in vacuum, calcined in air, and calcined in vacuum, respectively.

Jung et al. [42] have shown that vacuum annealing leads to a generation of n-donors in the form of oxygen vacancies, which results in increased emission at green wavelengths. In turn, the red PL from the recombination of electrons trapped on Ti^3+^ ions with valence band or shallow-trapped holes extends into the red emission with a maximum between 600 and 650 nm [18,33]. The most accurate deconvolution was performed using parameters shown in Table 3. Relative areas of Gauss peaks related to the green and red emissions are shown in Figure 8a,b. As it can be seen, green emission intensity of amorphous samples is enhanced due to the laser treatment in the air and vacuum. However, that effect is not observed for crystalline nanotubes. On the other hand, a red emission bands intensity decreases both for the as-anodized and calcined samples, with one exception, i.e., the laser modification of TiO_2_-30 led to the red emission increment. It is worth to note, that the lowest red emission is observed for the series of amorphous nanotubes after laser treatment in the vacuum. As seen in Figure 8b, the relative intensity of red PL band decreases for almost all samples due to the laser treatment. This phenomenon is related to the decrement of surface area that reduces the amount of chemisorbed O_2_ molecules quenching green PL. Thus, reduced surface area enhances green to red PL ratio.

## 4. Conclusions

In summary, we presented the impact of laser irradiation on titanium dioxide nanotubes morphology, structure, and optical properties, depending on the anodization time, crystallinity, and atmosphere. It was observed that, regardless of the structure of the titania material, laser processing leads to the formation of ca. 0.3 µm melted, re-solidified layer on the top of the nanotubes. However, the degradation degree strongly depends on the processing conditions and crystallinity of the samples. The best results in terms of preservation of the initial architecture of the NTs were obtained for calcined TiO_2_NTs that underwent laser treatment in air. From Raman measurements, it could be concluded that laser processing of the as-produced samples induces the phase conversion from amorphous to crystalline, whereas it reduces structure order of calcined ones. The grazing incident XRD measurements confirmed the phase transformation of amorphous material; however, for the sample obtained in the air, the top of the nanotube remains amorphous, and the anatase peak could not be detected for ca. 480 nm alongside the nanotube wall. In the case of calcined material, it could be deduced from XRD data that the process of crystal degradation may be initiated for vacuum treated samples. Although the morphology and crystal structure of the laser-processed titania nanotubes were degraded, the narrowing of the optical bandgap suggests that the prepared material could be further used in photoelectrochemical studies.

We would like to emphasize that such in-depth investigation of crystalline phase with the use of Gi-XRD indicating the crystallinity degree along the tubular layer of laser-treated amorphous and calcined TiO_2_ nanotubes was performed for the first time. Moreover, the laser processing combined with the usage of the motorized table could be easily scaled up to the commercial production level, and it allows for the modification of well-defined sample area that opens a way for many possible applications.

## Figures and Tables

**Table 1:** Summarization of all obtained samples regarding anodization time, thermal treatment, and atmosphere during laser treatment.

Atmosphere during Laser Treatment	Thermal Treatment	Anodization Time/min			
		15	30	45	60
air	as-anodized	TiO_2_-15	TiO_2_-30	TiO_2_-45	TiO_2_-60
	calcined	TiO_2_-15	TiO_2_-30	TiO_2_-45	TiO_2_-60
vacuum	as-anodized	TiO_2_-15	TiO_2_-30	TiO_2_-45	TiO_2_-60
	calcined	TiO_2_-15	TiO_2_-30	TiO_2_-45	TiO_2_-60

**Table 2:** Optical bandgap values determined from the Tauc plot curves shown in Figure 6.

Sample		Bandgap/eV	Sample		Bandgap/eV
reference as-anodized	TiO_2_-15	3.08	calcined laser-modified in air	TiO_2_-15	2.72
	TiO_2_-30	3.14		TiO_2_-30	2.77
	TiO_2_-45	3.15		TiO_2_-45	2.86
	TiO_2_-60	2.94		TiO_2_-60	2.9
reference calcined	TiO_2_-15	3.06	calcined laser-modified in vacuum	TiO_2_-15	2.62
	TiO_2_-30	3.08		TiO_2_-30	2.73
	TiO_2_-45	3.14		TiO_2_-45	2.78
	TiO_2_-60	2.73		TiO_2_-60	2.7

**Table 3:** The fitting parameters used to perform deconvolution of photoluminescence spectrum of TiO_2_-45.

Color	Peak Position (nm)	FWHM (nm)
blue	450	65
green	525	105
red 1	600	50
red 2	649	80
IR	733	20
